# Non-geneticist champions are essential to the mainstreaming of genomic medicine

**DOI:** 10.1038/s41431-024-01780-y

**Published:** 2025-01-03

**Authors:** Michael P. Mackley, Emma Weisz, Robin Z. Hayeems, Clara Gaff, Belinda Dawson-McClaren

**Affiliations:** 1 Division of Clinical & Metabolic Genetics, Department of Paediatrics, The Hospital for Sick Children, Toronto, ON Canada; 2 Department of General Paediatrics, Monash Children’s Hospital, Melbourne, VIC Australia; 3 Melbourne Genomics Health Alliance, Walter and Eliza Hall Institute, Melbourne, VIC Australia; 4 Program in Child Health Evaluative Sciences, SickKids Research Institute, Toronto, ON Canada; 5 Institute of Health Policy, Management and Evaluation, University of Toronto, Toronto, ON Canada; 6 Genomics in Society, Murdoch Children’s Research Institute, Melbourne, VIC Australia; 7 Department of Paediatrics, University of Melbourne, Melbourne, VIC Australia

**Keywords:** Genetic services, Genetic testing, Health policy, Genetic testing, Medical genomics

Demand for genomic testing is increasing across medicine as it paves the way for earlier diagnoses and targeted management of patients with rare diseases. The diagnostic utility of genomic testing has been clearly established, with evidence demonstrating value of its earlier positioning in the genetic care pathway [1, 2]. However, clinical genetics services are experiencing unsustainable pressures and are challenged to meet this growing need [3, 4]. In response, the roles of genetics professionals are shifting, with more working in spaces outside of the genetics service [5–7]. Recent studies have also illustrated an additional solution: greater involvement of non-geneticist clinicians in the delivery of genomic medicine [8, 9]. Concurrently, institutions and governments are becoming increasingly aware of the potential benefits of such “mainstreaming”—where non-geneticist clinicians are responsible for components of the genetic care pathway—and are supporting expanded and earlier access to these important tests [10]. To increase access, however, intentional and purpose-built strategies are needed to increase capacity and facilitate adoption into the practice of non-geneticist clinicians.

The authors of this piece are health services researchers and clinicians engaged in such efforts in their respective jurisdictions, working to broaden patient access to genomic testing through increased involvement of non-geneticist clinicians. In both Victoria, Australia, and Ontario, Canada, access to genomic testing is expanding beyond the clinical genetics service to include pediatricians and sub-specialists facilitating testing for eligible patients. In both jurisdictions genomic testing is available as a publicly funded test—this comes with a shared responsibility between the laboratories and government around resource allocation and affords a centralized approach to adjudicating eligibility for access. Rigorous health technology assessment underpins this decision-making, and eligibility criteria are attached to the test. In both Victoria and Ontario capacity has been built to perform this test locally and is being scaled to meet expected demand. Comparison of mainstreaming initiatives across settings affords a greater understanding of generalizable challenges and related solutions. These lessons can, in turn, optimize approaches to mainstreaming initiatives required to expand access to genomic testing.

Many challenges are shared across these jurisdictions. Although the specific details differ, in both settings there is a structural mismatch between the genetics clinic and pediatrics clinic—the latter is not structured or remunerated in a manner that easily facilitates the necessary components of the genomic testing process. These components include, but are not limited to: ability to organize testing for parents, longer length and flexibility of appointments to facilitate counselling, and administrative support. Furthermore, in both jurisdictions, and well-described in the literature [11], there is a perception of limited skill amongst non-geneticist clinicians that must be overcome. Relatedly, as clinical genetics services are often siloed in academic or tertiary centres, there is a decreasing familiarity with genetics services as one moves further away from these centres. This impacts non-geneticist clinicians’ connection to the genetics service and their comfort, willingness, and readiness to increase their scope of practice to include genomic testing and counselling.

Although diverse solutions have been proposed to overcome these barriers, the authors have observed the immense and far-reaching value of one strategy: genomics champions. Genomics champions are healthcare professionals working outside of the clinical genetics service who have a special interest in genomics and readily share their expertise with others [11]. Well-described in implementation science literature as a possible strategy [12], and with documented successes in genomics [13], it is our experience that champions are instrumental to reducing many of the barriers described above. The reality of this work is that systems cannot be dismantled and rebuilt in a genomics-friendly manner, and we must work within the systems that currently exist, hoping that these may change over time. Champions, by arising from and existing within specialties outside of the clinical genetics service, act as a bridge between these often-disconnected settings [13]. Champions can speak the language of both groups. As a result, they help both the genetics service and non-geneticist clinicians arrive at a place of shared value by understanding the priorities of both groups. This helps create space for genomics in an otherwise busy department by aligning it with the values and processes of that setting.

The authors have observed how champions can help address the shared challenges listed above. For instance, champions can work within the structural mismatch between practice settings both through understanding the intricacies of the disparate settings and through their willingness to work outside of conventional structures. Of note, this work is often initially over-and-above their expected clinical workloads. When champions establish relationships with the clinical genetics service, they bring these relationships into the spaces in which they routinely work, building networks for their colleagues. Furthermore, by working in non-genetics spaces, they provide an invaluable educational role, upskilling their colleagues in an iterative and experiential manner. They are also positioned to advocate for genomics at decision-making tables where the clinical genetics service is not represented.

If mainstreaming initiatives are to be successful, it is therefore essential that the role of the champion is at the forefront. Consideration must be given to identifying and developing genomics champions as well as supporting them in an intentional manner. This must occur at the level of clinical services, hospitals and institutions, as well as governing bodies and health systems.

At the level of clinical services, champions need to be embraced and intentionally supported; in other words, genomic champions need to be championed. Genetics services, often siloed in academic centres and pediatric hospitals, must invite champions to the table. Champions should be viewed as allies who extend the reach of the genetics service, providing care that the service cannot provide alone. Champions should be invited to relevant meetings, engaged deliberately and upskilled in an experiential manner. Efforts should be made to provide them with networks and resources they can share with colleagues. Services should endeavour to be accessible to champions, should questions arise or support be required. In the other direction, it is critical that genetics services provide intentional outreach and point of care feedback to champions to help develop their skills and knowledge.

At the level of hospitals and institutions, the implementation of structures that allow champions to work at the top of their growing scope is essential. The role of the champion within departments must be recognized and promoted to raise the genomics profile of the department and positively impact the champion’s colleagues. Clinical structures that facilitate the champion role—which can require additional administrative burdens and tasks that do not historically fit within the workflow of non-genetics clinics—must be developed.

National and international organizations with a mandate including genomic medicine must also turn their attention to the value of genomics champions. There will be a role for such organizations to support the upskilling and even credentialling of non-geneticist clinicians in genomic medicine. For example, colleges could promote cross-training or develop pathways to enable credentialling of non-geneticist clinicians in genomics or establish minimum competencies for non-geneticists engaged in this work.

These strategies must be coupled with allocation of resources to support non-geneticist clinicians filling this important role as part of health system implementation efforts. Both champions, and the structures needed to support their work, need to be considered in funding decisions—the success and accountability of champions is maximised when they are funded and protected to carry out this work. Importantly, funding of champions alone does not guarantee success and must be accompanied by the support and capacity building described above, as well as clear goals to ensure impact in their workplaces. Similarly, clinical genetics services must be resourced in a manner that allows them to support champions. Consideration must be given to measuring impact of genetics services in ways that are not direct patient care by geneticists—through supporting a network of champions, the genetics service can ultimately support more patients and protect clinical time for the complex patients who require their subspecialized care the most. Finally, in our experience, the champions who are most successful are those who are committed to the broader goal of expanding access to genomic testing and invested in filling the champion role in all its facets, including increasing capacity of all of those around them. Efforts should be made to identify the best individuals to help carry out this mission.

Ultimately, if the population at large is to benefit from advances in genomic medicine, genetics services cannot do this alone. Mainstreaming initiatives, where the delivery of genomic testing relies on non-geneticist clinicians to expand patient access, are becoming increasingly common and must continue to expand. The authors firmly believe that champions are central to the success and sustainability of these programs, and the future of genomic medicine at scale. As such, whether mainstreaming programs are being designed in a research-driven way or are grassroots initiatives rolled out by eager departments, the role of champions must be embraced, supported, and resourced accordingly. Without champions, the health system will struggle to truly enter the genomics era.
